# The genome sequence of *Dolichopus griseipennis *Stannius, 1831

**DOI:** 10.12688/wellcomeopenres.23382.1

**Published:** 2025-01-27

**Authors:** Mike Ashworth, James McCulloch, Liam M. Crowley, C. Martin Drake, Natural History Museum Genome Acquisition Lab, University of Oxford and Wytham Woods Genome Acquisition Lab, Darwin Tree of Life Barcoding collective, Wellcome Sanger Institute Tree of Life Management, Samples and Laboratory team, Wellcome Sanger Institute Scientific Operations: Sequencing Operations, Wellcome Sanger Institute Tree of Life Core Informatics team, Tree of Life Core Informatics collective, Darwin Tree of Life Consortium

**Affiliations:** 1 Independent researcher, Yeovil, England, UK; 2 University of Oxford, Oxford, England, UK; 3 Wellcome Sanger Institute, Hinxton, England, UK; 4 Independent researcher, Axminster, England, UK

**Keywords:** Dolichopus griseipennis, Dolichopodid fly, genome sequence, chromosomal, Diptera

## Abstract

We present a genome assembly from an individual male *Dolichopus griseipennis* (Arthropoda; Insecta; Diptera; Dolichopodidae). The genome sequence has a total length of 897.50 megabases. Most of the assembly is scaffolded into 6 chromosomal pseudomolecules, including the X sex chromosome. The mitochondrial genome has also been assembled and is 16.12 kilobases in length. Gene annotation of this assembly on Ensembl identified 12,532 protein-coding genes.

## Species taxonomy

Eukaryota; Opisthokonta; Metazoa; Eumetazoa; Bilateria; Protostomia; Ecdysozoa; Panarthropoda; Arthropoda; Mandibulata; Pancrustacea; Hexapoda; Insecta; Dicondylia; Pterygota; Neoptera; Endopterygota; Diptera; Brachycera; Muscomorpha; Eremoneura; Empidoidea; Dolichopodidae; Dolichopodinae; *Dolichopus*; *Dolichopus griseipennis* Stannius, 1831 (NCBI:txid327083).

## Background

The long-legged flies, family Dolichopodidae, are small to medium-sized flies. Those in the traditional family Dolichopodidae sensu stricto have some to many of the following characteristics: long, slender legs sometimes with adornments and protuberances in the males, in some genera raptorial, body slender, metallic green or bronze, sometimes with yellow markings, large prominent eyes, antennae with a long apical or dorsal arista, wings having simple venation with only one obvious crossvein (dm-m) placed beyond the basal third of the wing and emitting only two veins, the costa ending at or before vein M _1_, and other crossveins and cells very short and confined the extreme base of the wing. The male terminalia rotated and lateroflexed to the right. The basal subfamilies Microphorinae and Parathalassiinae are small dull brown or black species with slightly more complete venation ( Yang *et al.*, 2006).

Dolichopodids stand with an erect posture and can run quickly in pursuit of their prey or in search of a mate. Most are predatory in the adult and larval stages, feeding on small invertebrates. The larvae of *Dolichopus* have been recorded preying on small oligochaetes and the aquatic larvae of several nematoceran families such as mosquitos, chironomids and ceratopogonids ( Ulrich, 2004). Some species have complex courtship involving wing- or leg-waving and limited dancing. They occur in a wide range of habitats often favouring wet places including the margins of water bodies, mostly freshwater, though some species inhabit saline habitats.

*Dolichopus* Latreille, 1809 is the largest genus in the family with approaching 600 species worldwide ( Yang *et al.*, 2006). The genus is found in most biogeographic realms although absent from the Neotropical and Antarctic ( Brooks, 2005). Among British *Dolichopus* species with yellow femora and a ventral fringe on the hind femora, *Dolichopus griseipennis* Stannius, 1831 is the only species with a tiny hang-vein at the inflexion of vein M _1_; *nitidus* and *diadema* also have such a hang-vein but no femoral fringe. It differs from other morphologically similar species in having a long fine posteroventral seta on the front tibia and is one of the few species of *Dolichopus* with a single seta on the hind basitarsus. Females have the unique combination of the square bend and hang-vein on vein M _1_ and only one dorsal seta on the hind basitarsus. Rare specimens of *signifer* have a tiny hang-vein and one seta on the hind basitarsus and would run to *griseipennis* in the keys by d’Assis-Fonseca (1978) and Grichanov (2006) but they have dark front coxae. *D. griseipennis* appears to have a larger size range than found in most *Dolichopus*. The mean and range of the wing length are 5.3 (4.7–5.7) mm in males and females 5.1 (3.9–5.9) mm in females.

*Dolichopus griseipennis* is primarily a northern European species, with most records from Britain, France, Benelux and Scandinavia, but extending as far south as Portugal and Spain ( GBIF Secretariat, 2024). In Britain the distribution is predominantly southern, where it is common in much of England north to Yorkshire, although not in East Anglia, and infrequent in inland Wales, northern England and Scotland. However, it is distinguished by being found on Scottish islands including the Shetlands, Outer Hebrides and the tiny outlying North Rona and Sule Skerry. Presumably winter minimum temperature controls this distribution. It occurs with no apparent preference in a wide range of habitats, shaded to open, wet to moderately dry. The flight period is long, from April to November, peaking in July to early August.

A male *Dolichopus griseipennis* was taken on 15 July 2020 in a rural garden in Somerset, SW England. The specimen was sent live to the Natural History Museum, London. This species is one of the *Dolichopus* that can be found in drier-than-average sites, for a dolichopodid, along with *festivus*, *trivialis* and *virgultorum*, and is not uncommon in rural garden situations.

The generation of a high-quality genome sequence for *Dolichopus griseipennis* is an important step in advancing the understanding of these fascinating flies and in determining relationships between species within the genus *Dolichopus* and within the Dolichopodidae.

## Genome sequence report

**Figure 1.**
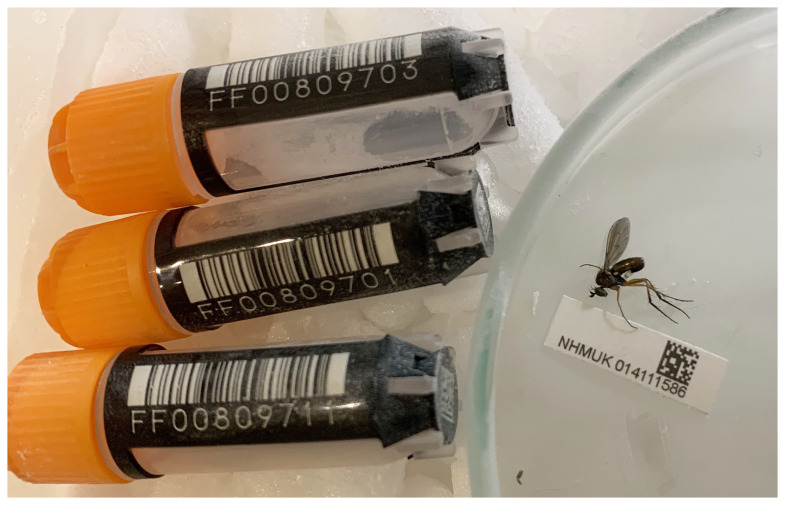
Photograph of the *Dolichopus griseipennis* (idDolGris1) specimen used for genome sequencing.

The genome of an adult male *Dolichopus griseipennis* ( Figure 1) was sequenced using Pacific Biosciences single-molecule HiFi long reads, generating a total of 24.18 Gb (gigabases) from 2.70 million reads, providing approximately 34-fold coverage. Primary assembly contigs were scaffolded with chromosome conformation Hi-C data, which produced 104.44 Gb from 691.64 million reads. Specimen and sequencing details are provided in Table 1.

**Table 1.** Specimen and sequencing data for *Dolichopus griseipennis*.

Project information			
**Study title**	*Dolichopus griseipennis*		
**Umbrella BioProject**	PRJEB60317		
**Species**	*Dolichopus griseipennis*		
**BioSample**	SAMEA7521634		
**NCBI taxonomy ID**	327083		
Specimen information			
**Technology**	**ToLID**	**BioSample accession**	**Organism part**
**PacBio long read sequencing**	idDolGris1	SAMEA7521646	Thorax
**Hi-C sequencing**	idDolGris1	SAMEA7521645	Head
**RNA sequencing**	idDolGris2	SAMEA112232980	Whole organism
Sequencing information			
**Platform**	**Run accession**	**Read count**	**Base count (Gb)**
**Hi-C Illumina NovaSeq 6000**	ERR10968299	6.92e+08	104.44
**PacBio Sequel IIe**	ERR10962213	2.70e+06	24.18
**RNA Illumina NovaSeq 6000**	ERR11242533	6.56e+07	9.9

**Figure 2.**
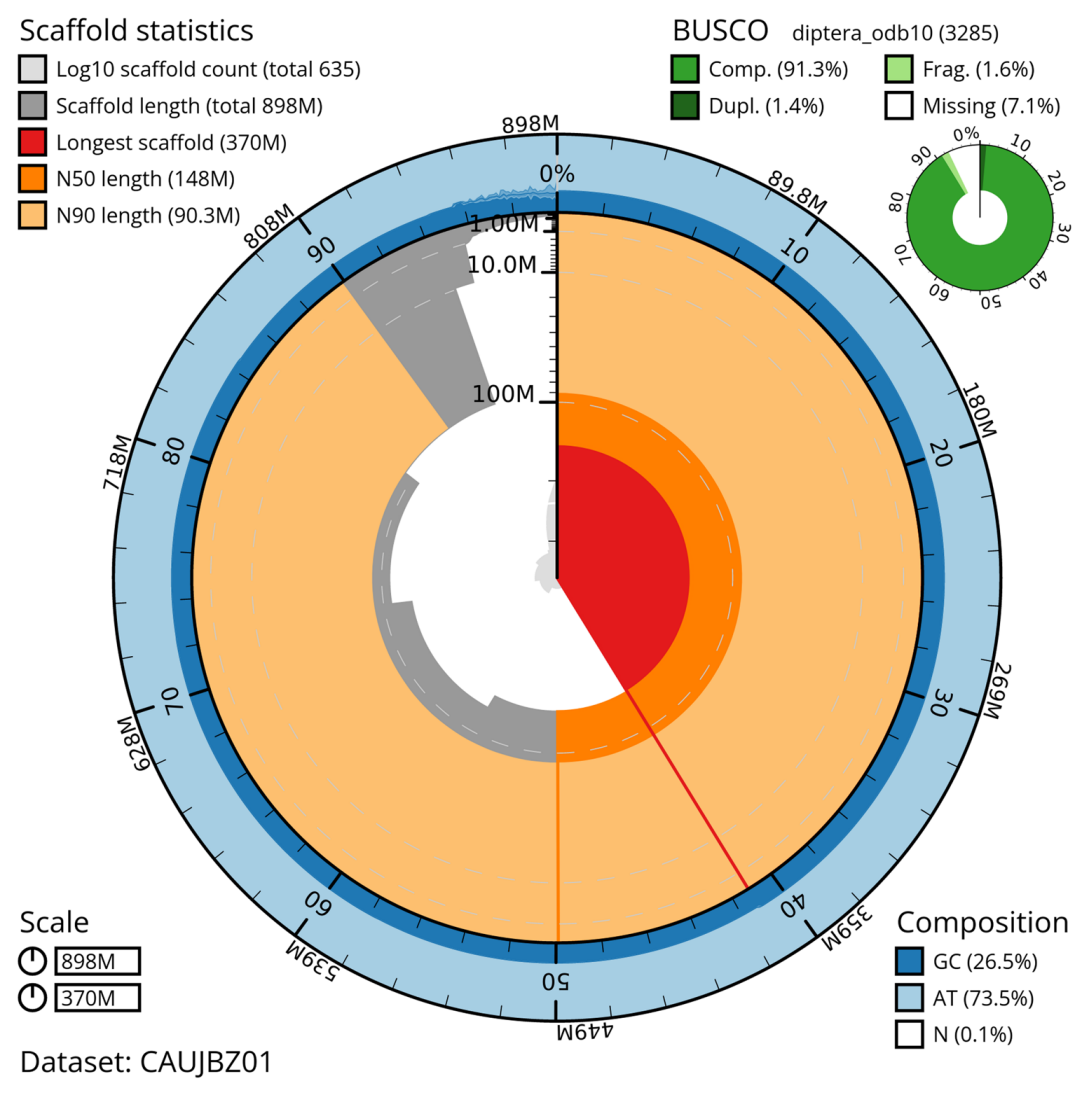
Genome assembly of *Dolichopus griseipennis*, idDolGris1.1: metrics. The BlobToolKit snail plot shows N50 metrics and BUSCO gene completeness. The main plot is divided into 1,000 bins around the circumference with each bin representing 0.1% of the 897,543,505 bp assembly. The distribution of scaffold lengths is shown in dark grey with the plot radius scaled to the longest scaffold present in the assembly (370,326,948 bp, shown in red). Orange and pale-orange arcs show the N50 and N90 scaffold lengths (148,407,772 and 90,323,934 bp), respectively. The pale grey spiral shows the cumulative scaffold count on a log scale with white scale lines showing successive orders of magnitude. The blue and pale-blue area around the outside of the plot shows the distribution of GC, AT and N percentages in the same bins as the inner plot. A summary of complete, fragmented, duplicated and missing BUSCO genes in the diptera_odb10 set is shown in the top right. An interactive version of this figure is available at https://blobtoolkit.genomehubs.org/view/CAUJBZ01/dataset/CAUJBZ01/snail.

**Figure 3.**
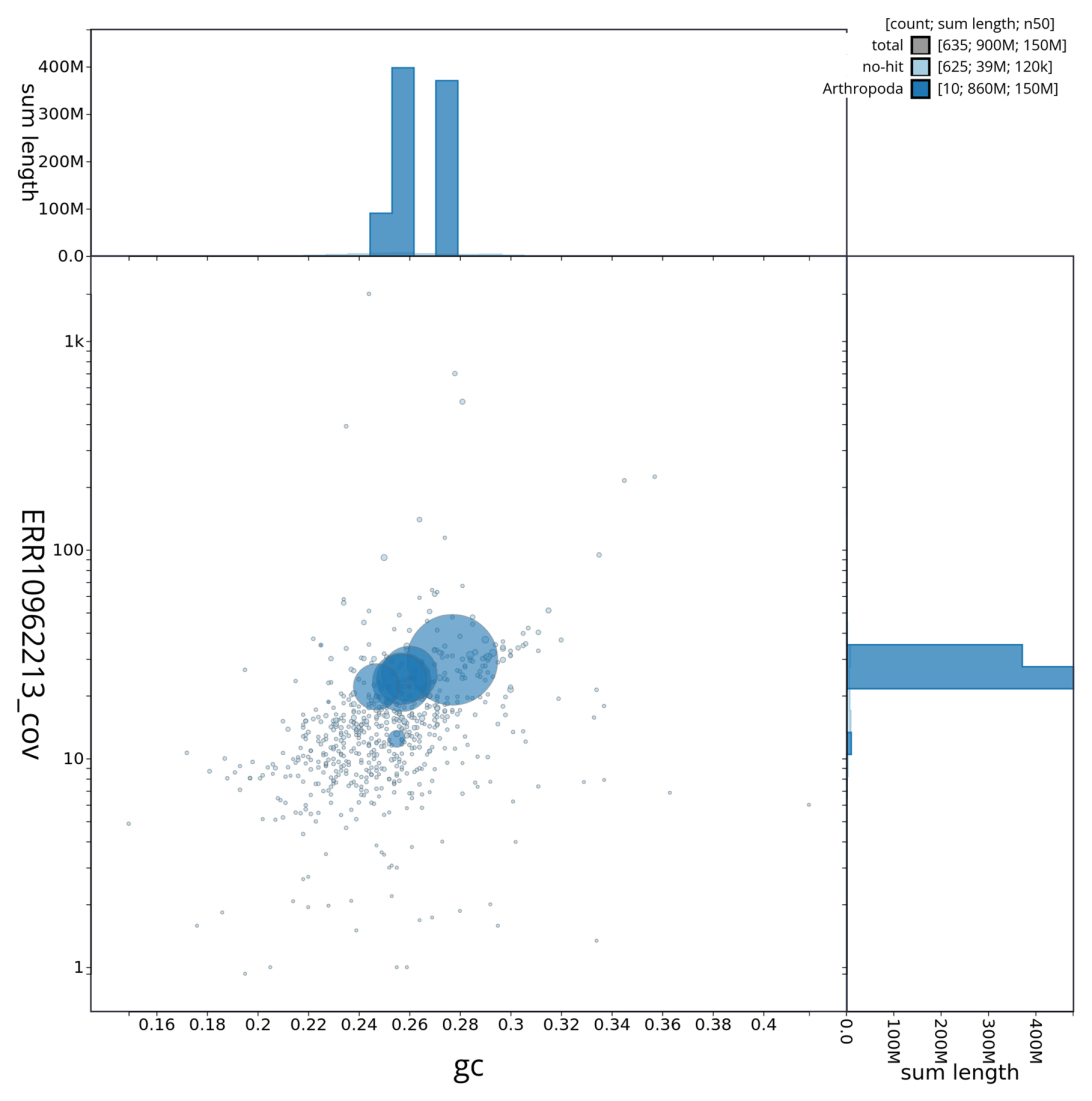
Genome assembly of *Dolichopus griseipennis*, idDolGris1.1: BlobToolKit GC-coverage plot. Sequences are coloured by phylum. Circles are sized in proportion to sequence length. Histograms show the distribution of sequence length sum along each axis. An interactive version of this figure is available at https://blobtoolkit.genomehubs.org/view/CAUJBZ01/dataset/CAUJBZ01/blob.

**Figure 4.**
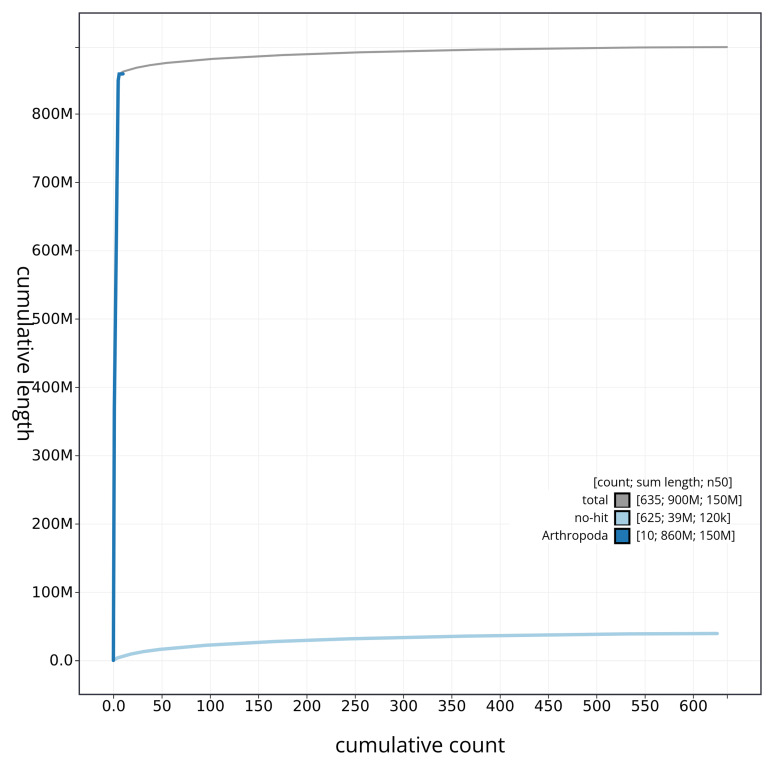
Genome assembly of *Dolichopus griseipennis* idDolGris1.1: BlobToolKit cumulative sequence plot. The grey line shows cumulative length for all sequences. Coloured lines show cumulative lengths of sequences assigned to each phylum using the buscogenes taxrule. An interactive version of this figure is available at https://blobtoolkit.genomehubs.org/view/CAUJBZ01/dataset/CAUJBZ01/cumulative.

**Figure 5.**
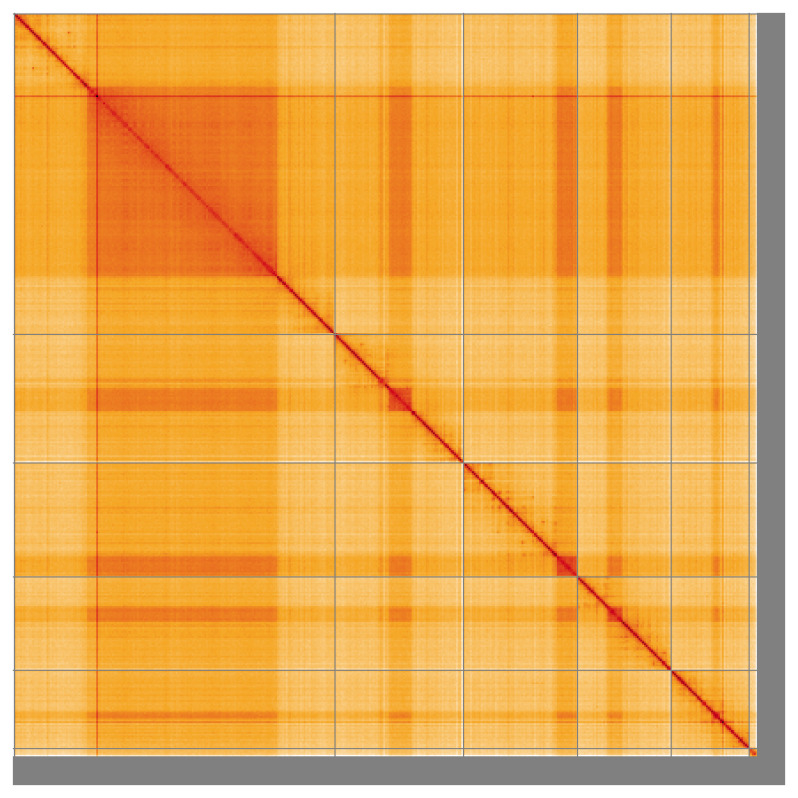
Genome assembly of *Dolichopus griseipennis* idDolGris1.1: Hi-C contact map of the idDolGris1.1 assembly, visualised using HiGlass. Chromosomes are shown in order of size from left to right and top to bottom. An interactive version of this figure may be viewed at https://genome-note-higlass.tol.sanger.ac.uk/l/?d=HsNzSoBGR_205GbucHg4wg.

Manual assembly curation corrected 164 missing joins or mis-joins and 12 haplotypic duplications, reducing the assembly length by 0.68% and the scaffold number by 14.42%, and increasing the scaffold N50 by 28.76%. The final assembly has a total length of 897.50 Mb in 634 sequence scaffolds with a scaffold N50 of 148.4 Mb ( Table 2). The total count of gaps in the scaffolds is 2,672. The snail plot in Figure 2 provides a summary of the assembly statistics, while the distribution of assembly scaffolds on GC proportion and coverage is shown in Figure 3. The cumulative assembly plot in Figure 4 shows curves for subsets of scaffolds assigned to different phyla. Most (96.13%) of the assembly sequence was assigned to 6 chromosomal-level scaffolds, representing 5 autosomes and the X sex chromosome. Chromosome-scale scaffolds confirmed by the Hi-C data are named in order of size ( Figure 5; Table 3). Chromosome X was assigned based on read coverage statistics. However, no Y sequence could be identified, and the species may be an XO male. The region of Chromosome 1 from ~83–305 Mb is of uncertain order and orientation. While not fully phased, the assembly deposited is of one haplotype. Contigs corresponding to the second haplotype have also been deposited. The mitochondrial genome was also assembled and can be found as a contig within the multifasta file of the genome submission.

**Table 2.** Genome assembly data for *Dolichopus griseipennis*, idDolGris1.1.

Genome assembly		
Assembly name	idDolGris1.1	
Assembly accession	GCA_963082915.1	
*Accession of alternate haplotype*	*GCA_963082765.1*	
Span (Mb)	897.50	
Number of contigs	3,307	
Contig N50 length (Mb)	0.5	
Number of scaffolds	634	
Scaffold N50 length (Mb)	148.4	
Longest scaffold (Mb)	370.33	
Assembly metrics *		*Benchmark*
Consensus quality (QV)	56.9	*≥ 50*
*k*-mer completeness	99.99%	*≥ 95%*
BUSCO **	C:91.3%[S:89.9%,D:1.4%], F:1.6%,M:7.1%,n:3,285	*C ≥ 95%*
Percentage of assembly mapped to chromosomes	96.13%	*≥ 95%*
Sex chromosomes	X	*localised homologous pairs*
Organelles	Mitochondrial genome: 16.12 kb	*complete single alleles*
Genome annotation of assembly GCA_963082915.1 at Ensembl		
Number of protein-coding genes	12,532	
Number of non-coding genes	2,113	
Number of gene transcripts	21,140	

* Assembly metric benchmarks are adapted from column VGP-2020 of “Table 1: Proposed standards and metrics for defining genome assembly quality” from Rhie *et al.* (2021).

** BUSCO scores based on the diptera_odb10 BUSCO set using version 5.3.2. C = complete [S = single copy, D = duplicated], F = fragmented, M = missing, n = number of orthologues in comparison. A full set of BUSCO scores is available at https://blobtoolkit.genomehubs.org/view/CAUJBZ01/dataset/CAUJBZ01/busco.

**Table 3.** Chromosomal pseudomolecules in the genome assembly of *Dolichopus griseipennis*, idDolGris1.

**INSDC accession**	**Name**	**Length (Mb)**	**GC%**
OY720441.1	1	370.33	27.5
OY720442.1	2	148.41	25.5
OY720443.1	3	131.55	26.0
OY720444.1	4	108.16	25.5
OY720445.1	5	90.32	24.5
OY720446.1	X	9.35	25.5
OY720447.1	MT	0.02	25.0

The estimated Quality Value (QV) of the final assembly is 56.9 with *k*-mer completeness of 99.99%, and the assembly has a BUSCO v5.3.2 completeness of 91.3% (single = 89.9%, duplicated = 1.4%), using the diptera_odb10 reference set ( *n* = 3,285).

Metadata for specimens, BOLD barcode results, spectra estimates, sequencing runs, contaminants and pre-curation assembly statistics are given at https://links.tol.sanger.ac.uk/species/327083.

## Genome annotation report

The *Dolichopus griseipennis* genome assembly (GCA_963082915.1) was annotated at the European Bioinformatics Institute (EBI) on Ensembl Rapid Release. The resulting annotation includes 21,140 transcribed mRNAs from 12,532 protein-coding and 2,113 non-coding genes ( Table 2; https://rapid.ensembl.org/Dolichopus_griseipennis_GCA_963082915.1/Info/Index). The average transcript length is 14,401.78. There are 1.44 coding transcripts per gene and 5.21 exons per transcript.

## Methods

### Sample acquisition and DNA barcoding

An adult male specimen of *Dolichopus griseipennis* (specimen ID NHMUK014111586, ToLID idDolGris1) was collected from Yeovil, England, UK (latitude 50.97, longitude –2.68) on 2020-07-15, using an aerial net. The specimen was collected and identified by Mike Ashworth (independent researcher) and preserved on dry ice.

The specimen used for RNA sequencing (specimen ID Ox002300, ToLID idDolGris2) was an adult specimen collected from Wytham Woods, Oxfordshire (biological vice-count (latitude 51.77, longitude –1.34) on 2022-07-05. The specimen was collected by James McCulloch and Liam Crowley (University of Oxford), identified by James McCulloch and then preserved on dry ice.

The initial identification was verified by an additional DNA barcoding process according to the framework developed by Twyford *et al*. (2024). A small sample was dissected from the specimens and stored in ethanol, while the remaining parts of the specimen were shipped on dry ice to the Wellcome Sanger Institute (WSI). The tissue was lysed, the COI marker region was amplified by PCR, and amplicons were sequenced and compared to the BOLD database, confirming the species identification ( Crowley *et al.*, 2023). Following whole genome sequence generation, the relevant DNA barcode region is also used alongside the initial barcoding data for sample tracking at the WSI ( Twyford *et al.*, 2024). The standard operating procedures for Darwin Tree of Life barcoding have been deposited on protocols.io ( Beasley *et al.*, 2023).

### Nucleic acid extraction

The workflow for high molecular weight (HMW) DNA extraction at the Wellcome Sanger Institute (WSI) Tree of Life Core Laboratory includes a sequence of core procedures: sample preparation and homogenisation, DNA extraction, fragmentation and purification. Detailed protocols are available on protocols.io ( Denton *et al.*, 2023b). The idDolGris1 sample was weighed and dissected on dry ice ( Jay *et al.*, 2023), and tissue from the thorax was homogenised using a PowerMasher II tissue disruptor ( Denton *et al.*, 2023a). HMW DNA was extracted using the Manual MagAttract v1 protocol ( Strickland *et al.*, 2023b). DNA was sheared into an average fragment size of 12–20 kb in a Megaruptor 3 system ( Todorovic *et al.*, 2023). Sheared DNA was purified by solid-phase reversible immobilisation, using AMPure PB beads to eliminate shorter fragments and concentrate the DNA ( Strickland *et al.*, 2023a). The concentration of the sheared and purified DNA was assessed using a Nanodrop spectrophotometer and Qubit Fluorometer using the Qubit dsDNA High Sensitivity Assay kit. Fragment size distribution was evaluated by running the sample on the FemtoPulse system.

RNA was extracted from whole organism tissue of idDolGris2 in the Tree of Life Laboratory at the WSI using the RNA Extraction: Automated MagMax™ *mir*Vana protocol ( do Amaral *et al.*, 2023). The RNA concentration was assessed using a Nanodrop spectrophotometer and a Qubit Fluorometer using the Qubit RNA Broad-Range Assay kit. Analysis of the integrity of the RNA was done using the Agilent RNA 6000 Pico Kit and Eukaryotic Total RNA assay.

### Hi-C preparation

Head tissue of the idDolGris1 sample was processed using the Arima-HiC v2 kit. In brief, frozen tissue (stored at –80 °C) was fixed, and the DNA crosslinked using a TC buffer with 22% formaldehyde. After crosslinking, the tissue was homogenised using the Diagnocine Power Masher-II and BioMasher-II tubes and pestles. Following the kit manufacturer's instructions, crosslinked DNA was digested using a restriction enzyme master mix. The 5’-overhangs were then filled in and labelled with biotinylated nucleotides and proximally ligated. An overnight incubation was carried out for enzymes to digest remaining proteins and for crosslinks to reverse. A clean up was performed with SPRIselect beads prior to library preparation.

### Library preparation and sequencing

Library preparation and sequencing were performed at the WSI Scientific Operations core. Pacific Biosciences HiFi circular consensus DNA sequencing libraries were prepared using the PacBio Express Template Preparation Kit v2.0 (Pacific Biosciences, California, USA) as per the manufacturer’s instructions. The kit includes the reagents required for removal of single-strand overhangs, DNA damage repair, end repair/A-tailing, adapter ligation, and nuclease treatment. Library preparation also included a library purification step using 0.8X AMPure PB beads and a size selection step to remove templates < 3 kb using AMPure PB modified SPRI. Samples were sequenced using the Sequel IIe system (Pacific Biosciences, California, USA). The concentration of the library loaded onto the Sequel IIe was within the manufacturer's recommended loading concentration range of 40–135 pM. The SMRT link software, a PacBio web-based end-to-end workflow manager, was used to set-up and monitor the run, as well as perform primary and secondary analysis of the data upon completion.

For Hi-C library preparation, DNA was fragmented to a size of 400 to 600 bp using a Covaris E220 sonicator. The DNA was then enriched, barcoded, and amplified using the NEBNext Ultra II DNA Library Prep Kit following manufacturers’ instructions. The Hi-C sequencing was performed using paired-end sequencing with a read length of 150 bp on an Illumina NovaSeq 6000 instrument.

Poly(A) RNA-Seq libraries were constructed using the NEB Ultra II RNA Library Prep kit, following the manufacturer’s instructions. RNA sequencing was performed on the Illumina NovaSeq 6000 instrument.

### Genome assembly, curation and evaluation

***Assembly***

The HiFi reads were first assembled using Hifiasm ( Cheng *et al.*, 2021) with the --primary option. Haplotypic duplications were identified and removed using purge_dups ( Guan *et al.*, 2020). The Hi-C reads were mapped to the primary contigs using bwa-mem2 ( Vasimuddin *et al.*, 2019). The contigs were further scaffolded using the provided Hi-C data ( Rao *et al.*, 2014) in YaHS ( Zhou *et al.*, 2023) using the --break option. The scaffolded assemblies were evaluated using Gfastats ( Formenti *et al.*, 2022), BUSCO ( Manni *et al.*, 2021) and MERQURY.FK ( Rhie *et al.*, 2020).

The mitochondrial genome was assembled using MitoHiFi ( Uliano-Silva *et al.*, 2023), which runs MitoFinder ( Allio *et al.*, 2020) and uses these annotations to select the final mitochondrial contig and to ensure the general quality of the sequence.

***Assembly curation***

The assembly was decontaminated using the Assembly Screen for Cobionts and Contaminants (ASCC) pipeline (article in preparation). Manual curation was primarily conducted using PretextView ( Harry, 2022), with additional insights provided by JBrowse2 ( Diesh *et al.*, 2023) and HiGlass ( Kerpedjiev *et al.*, 2018). Scaffolds were visually inspected and corrected as described by Howe *et al*. (2021). Any identified contamination, missed joins, and mis-joins were corrected, and duplicate sequences were tagged and removed. The entire process is documented at https://gitlab.com/wtsi-grit/rapid-curation (article in preparation).

***Evaluation of the final assembly***

A Hi-C map for the final assembly was produced using bwa-mem2 ( Vasimuddin *et al.*, 2019) in the Cooler file format ( Abdennur & Mirny, 2020). To assess the assembly metrics, the *k*-mer completeness and QV consensus quality values were calculated in Merqury ( Rhie *et al.*, 2020). This work was done using the “sanger-tol/readmapping” ( Surana *et al.*, 2023a) and “sanger-tol/genomenote” ( Surana *et al.*, 2023b) pipelines. The genome evaluation pipelines were developed using nf-core tooling ( Ewels *et al.*, 2020) and MultiQC ( Ewels *et al.*, 2016), relying on the Conda package manager, the Bioconda initiative ( Grüning *et al.*, 2018), the Biocontainers infrastructure ( da Veiga Leprevost *et al.*, 2017), as well as the Docker ( Merkel, 2014) and Singularity ( Kurtzer *et al.*, 2017) containerisation solutions.

The genome was also analysed within the BlobToolKit environment ( Challis *et al.*, 2020) and BUSCO scores ( Manni *et al.*, 2021) were calculated.

Table 4 contains a list of relevant software tool versions and sources.

**Table 4.** Software tools: versions and sources.

**Software tool**	**Version**	**Source**
BlobToolKit	4.2.1	https://github.com/blobtoolkit/blobtoolkit
BUSCO	5.3.2	https://gitlab.com/ezlab/busco
bwa-mem2	2.2.1	https://github.com/bwa-mem2/bwa-mem2
Cooler	0.8.11	https://github.com/open2c/cooler
FastK	427104ea91c78c3b8b8b49f1a7d6bbeaa869ba1c	https://github.com/thegenemyers/FASTK
Hifiasm	0.16.1-r375	https://github.com/chhylp123/hifiasm
HiGlass	44086069ee7d4d3f6f3f0012569789ec138f42b84aa44357826c0b6753eb28de	https://github.com/higlass/higlass
Merqury.FK	d00d98157618f4e8d1a9190026b19b471055b22e	https://github.com/thegenemyers/MERQURY.FK
MitoHiFi	2	https://github.com/marcelauliano/MitoHiFi
Nextflow	23.04.0-5857	https://github.com/nextflow-io/nextflow
PretextView	0.2	https://github.com/wtsi-hpag/PretextView
purge_dups	1.2.3	https://github.com/dfguan/purge_dups
sanger-tol/ascc	-	https://github.com/sanger-tol/ascc
sanger-tol/genomenote	v1.0	https://github.com/sanger-tol/genomenote
sanger-tol/readmapping	1.1.0	https://github.com/sanger-tol/readmapping/tree/1.1.0
YaHS	1.2a	https://github.com/c-zhou/yahs

### Genome annotation

The Ensembl Genebuild annotation system ( Aken *et al.*, 2016) was used to generate annotation for the *Dolichopus griseipennis* assembly (GCA_963082915.1) in Ensembl Rapid Release at the EBI. Annotation was created primarily through alignment of transcriptomic data to the genome, with gap filling via protein-to-genome alignments of a select set of proteins from UniProt ( UniProt Consortium, 2019).

### Wellcome Sanger Institute – Legal and Governance

The materials that have contributed to this genome note have been supplied by a Darwin Tree of Life Partner. The submission of materials by a Darwin Tree of Life Partner is subject to the **‘Darwin Tree of Life Project Sampling Code of Practice’**, which can be found in full on the Darwin Tree of Life website here. By agreeing with and signing up to the Sampling Code of Practice, the Darwin Tree of Life Partner agrees they will meet the legal and ethical requirements and standards set out within this document in respect of all samples acquired for, and supplied to, the Darwin Tree of Life Project.

Further, the Wellcome Sanger Institute employs a process whereby due diligence is carried out proportionate to the nature of the materials themselves, and the circumstances under which they have been/are to be collected and provided for use. The purpose of this is to address and mitigate any potential legal and/or ethical implications of receipt and use of the materials as part of the research project, and to ensure that in doing so we align with best practice wherever possible. The overarching areas of consideration are:

•    Ethical review of provenance and sourcing of the material

•    Legality of collection, transfer and use (national and international)

Each transfer of samples is further undertaken according to a Research Collaboration Agreement or Material Transfer Agreement entered into by the Darwin Tree of Life Partner, Genome Research Limited (operating as the Wellcome Sanger Institute), and in some circumstances other Darwin Tree of Life collaborators.

## Data Availability

European Nucleotide Archive: Dolichopus griseipennis. Accession number PRJEB60317; https://identifiers.org/ena.embl/PRJEB60317. The genome sequence is released openly for reuse. The *Dolichopus griseipennis* genome sequencing initiative is part of the Darwin Tree of Life (DToL) project. All raw sequence data and the assembly have been deposited in INSDC databases. Raw data and assembly accession identifiers are reported in Table 1 and Table 2.

## Author information

Members of the University of Oxford and Wytham Woods Genome Acquisition Lab are listed here: https://doi.org/10.5281/zenodo.12157525.

Members of the Natural History Museum Genome Acquisition Lab are listed here: https://doi.org/10.5281/zenodo.12159242.

Members of the Darwin Tree of Life Barcoding collective are listed here: https://doi.org/10.5281/zenodo.12158331

Members of the Wellcome Sanger Institute Tree of Life Management, Samples and Laboratory team are listed here: https://doi.org/10.5281/zenodo.12162482.

Members of Wellcome Sanger Institute Scientific Operations: Sequencing Operations are listed here: https://doi.org/10.5281/zenodo.12165051.

Members of the Wellcome Sanger Institute Tree of Life Core Informatics team are listed here: https://doi.org/10.5281/zenodo.12160324.

Members of the Tree of Life Core Informatics collective are listed here: https://doi.org/10.5281/zenodo.12205391.

Members of the Darwin Tree of Life Consortium are listed here: https://doi.org/10.5281/zenodo.4783558.

## References

[ref-1] Abdennur N Mirny LA : Cooler: scalable storage for Hi-C data and other genomically labeled arrays. *Bioinformatics.* 2020;36(1):311–316. 10.1093/bioinformatics/btz540 31290943 PMC8205516

[ref-2] Aken BL Ayling S Barrell D : The ensembl gene annotation system. *Database (Oxford).* 2016;2016: baw093. 10.1093/database/baw093 27337980 PMC4919035

[ref-3] Allio R Schomaker-Bastos A Romiguier J : MitoFinder: efficient automated large-scale extraction of mitogenomic data in target enrichment phylogenomics. *Mol Ecol Resour.* 2020;20(4):892–905. 10.1111/1755-0998.13160 32243090 PMC7497042

[ref-4] Beasley J Uhl R Forrest LL : DNA barcoding SOPs for the Darwin Tree of Life project. *protocols.io.* 2023; [Accessed 25 June 2024]. 10.17504/protocols.io.261ged91jv47/v1

[ref-5] Brooks SE : Systematics and phylogeny of Dolichopodinae (Diptera: Dolichopodidae). *Zootaxa.* 2005;857(1). 10.11646/zootaxa.857.1.1

[ref-6] Challis R Richards E Rajan J : BlobToolKit – interactive quality assessment of genome assemblies. *G3 (Bethesda).* 2020;10(4):1361–1374. 10.1534/g3.119.400908 32071071 PMC7144090

[ref-7] Cheng H Concepcion GT Feng X : Haplotype-resolved *de novo* assembly using phased assembly graphs with hifiasm. *Nat Methods.* 2021;18(2):170–175. 10.1038/s41592-020-01056-5 33526886 PMC7961889

[ref-8] Crowley L Allen H Barnes I : A sampling strategy for genome sequencing the British terrestrial arthropod fauna [version 1; peer review: 2 approved]. *Wellcome Open Res.* 2023;8:123. 10.12688/wellcomeopenres.18925.1 37408610 PMC10318377

[ref-9] da Veiga Leprevost F Grüning BA Alves Aflitos S : BioContainers: an open-source and community-driven framework for software standardization. *Bioinformatics.* 2017;33(16):2580–2582. 10.1093/bioinformatics/btx192 28379341 PMC5870671

[ref-10] d’Assis-Fonseca ECM : Diptera Orthorrhapha Brachycera Dolichopodidae.1978;9. Reference Source

[ref-11] Denton A Oatley G Cornwell C : Sanger Tree of Life sample homogenisation: PowerMash. *protocols.io.* 2023a. 10.17504/protocols.io.5qpvo3r19v4o/v1

[ref-12] Denton A Yatsenko H Jay J : Sanger Tree of Life wet laboratory protocol collection V.1. *protocols.io.* 2023b. 10.17504/protocols.io.8epv5xxy6g1b/v1

[ref-13] Diesh C Stevens GJ Xie P : JBrowse 2: a modular genome browser with views of synteny and structural variation. *Genome Biol.* 2023;24(1): 74. 10.1186/s13059-023-02914-z 37069644 PMC10108523

[ref-14] do Amaral RJV Bates A Denton A : Sanger Tree of Life RNA extraction: automated MagMax ^TM^ mirVana. *protocols.io.* 2023. 10.17504/protocols.io.6qpvr36n3vmk/v1

[ref-15] Ewels P Magnusson M Lundin S : MultiQC: summarize analysis results for multiple tools and samples in a single report. *Bioinformatics.* 2016;32(19):3047–3048. 10.1093/bioinformatics/btw354 27312411 PMC5039924

[ref-16] Ewels PA Peltzer A Fillinger S : The nf-core framework for community-curated bioinformatics pipelines. *Nat Biotechnol.* 2020;38(3):276–278. 10.1038/s41587-020-0439-x 32055031

[ref-17] Formenti G Abueg L Brajuka A : Gfastats: conversion, evaluation and manipulation of genome sequences using assembly graphs. *Bioinformatics.* 2022;38(17):4214–4216. 10.1093/bioinformatics/btac460 35799367 PMC9438950

[ref-18] GBIF Secretariat: *Dolichopus griseipennis* Stannius, 1831.2024. Reference Source

[ref-19] Grichanov IY : A checklist and keys to North European genera and species of Dolichopodidae (Diptera). St Petersburg. VIZR RAAS (Plant Protection News Supplement),2006. Reference Source

[ref-20] Grüning B Dale R Sjödin A : Bioconda: sustainable and comprehensive software distribution for the life sciences. *Nat Methods.* 2018;15(7):475–476. 10.1038/s41592-018-0046-7 29967506 PMC11070151

[ref-21] Guan D McCarthy SA Wood J : Identifying and removing haplotypic duplication in primary genome assemblies. *Bioinformatics.* 2020;36(9):2896–2898. 10.1093/bioinformatics/btaa025 31971576 PMC7203741

[ref-22] Harry E : PretextView (Paired REad TEXTure Viewer): a desktop application for viewing pretext contact maps.2022. Reference Source

[ref-23] Howe K Chow W Collins J : Significantly improving the quality of genome assemblies through curation. *GigaScience.* 2021;10(1): giaa153. 10.1093/gigascience/giaa153 33420778 PMC7794651

[ref-24] Jay J Yatsenko H Narváez-Gómez JP : Sanger Tree of Life sample preparation: triage and dissection. *protocols.io.* 2023. 10.17504/protocols.io.x54v9prmqg3e/v1

[ref-25] Kerpedjiev P Abdennur N Lekschas F : HiGlass: web-based visual exploration and analysis of genome interaction maps. *Genome Biol.* 2018;19(1): 125. 10.1186/s13059-018-1486-1 30143029 PMC6109259

[ref-26] Kurtzer GM Sochat V Bauer MW : Singularity: scientific containers for mobility of compute. *PLoS One.* 2017;12(5): e0177459. 10.1371/journal.pone.0177459 28494014 PMC5426675

[ref-27] Manni M Berkeley MR Seppey M : BUSCO update: novel and streamlined workflows along with broader and deeper phylogenetic coverage for scoring of eukaryotic, prokaryotic, and viral genomes. *Mol Biol Evol.* 2021;38(10):4647–4654. 10.1093/molbev/msab199 34320186 PMC8476166

[ref-28] Merkel D : Docker: lightweight Linux containers for consistent development and deployment. *Linux J.* 2014;2014(239): 2; [Accessed 2 April 2024]. Reference Source

[ref-29] Rao SSP Huntley MH Durand NC : A 3D map of the human genome at kilobase resolution reveals principles of chromatin looping. *Cell.* 2014;159(7):1665–1680. 10.1016/j.cell.2014.11.021 25497547 PMC5635824

[ref-30] Rhie A McCarthy SA Fedrigo O : Towards complete and error-free genome assemblies of all vertebrate species. *Nature.* 2021;592(7856):737–746. 10.1038/s41586-021-03451-0 33911273 PMC8081667

[ref-31] Rhie A Walenz BP Koren S : Merqury: reference-free quality, completeness, and phasing assessment for genome assemblies. *Genome Biol.* 2020;21(1): 245. 10.1186/s13059-020-02134-9 32928274 PMC7488777

[ref-32] Strickland M Cornwell C Howard C : Sanger Tree of Life fragmented DNA clean up: manual SPRI. *protocols.io.* 2023a. 10.17504/protocols.io.kxygx3y1dg8j/v1

[ref-33] Strickland M Moll R Cornwell C : Sanger Tree of Life HMW DNA extraction: manual MagAttract. *protocols.io.* 2023b. 10.17504/protocols.io.6qpvr33novmk/v1

[ref-34] Surana P Muffato M Qi G : sanger-tol/readmapping: sanger-tol/readmapping v1.1.0 - Hebridean Black (1.1.0). *Zenodo.* 2023a. 10.5281/zenodo.7755669

[ref-35] Surana P Muffato M Sadasivan Baby C : sanger-tol/genomenote (v1.0.dev). *Zenodo.* 2023b. 10.5281/zenodo.6785935

[ref-36] Todorovic M Sampaio F Howard C : Sanger Tree of Life HMW DNA fragmentation: diagenode Megaruptor ^®^3 for PacBio HiFi. *protocols.io.* 2023. 10.17504/protocols.io.8epv5x2zjg1b/v1

[ref-37] Twyford AD Beasley J Barnes I : A DNA barcoding framework for taxonomic verification in the Darwin Tree of Life project [version 1; peer review: awaiting peer review]. *Wellcome Open Res.* 2024;9:339. 10.12688/wellcomeopenres.21143.1 39386966 PMC11462125

[ref-38] Uliano-Silva M Ferreira JGRN Krasheninnikova K : MitoHiFi: a python pipeline for mitochondrial genome assembly from PacBio high fidelity reads. *BMC Bioinformatics.* 2023;24(1): 288. 10.1186/s12859-023-05385-y 37464285 PMC10354987

[ref-39] Ulrich H : Predation by adult Dolichopodidae (Diptera): a review of literature with an annotated prey-predator list. *Studia Dipterologica.* 2004;11(2):369–403. Reference Source

[ref-40] UniProt Consortium: UniProt: a worldwide hub of protein knowledge. *Nucleic Acids Res.* 2019;47(D1):D506–D515. 10.1093/nar/gky1049 30395287 PMC6323992

[ref-41] Vasimuddin M Misra S Li H : Efficient architecture-aware acceleration of BWA-MEM for multicore systems.In: *2019 IEEE International Parallel and Distributed Processing Symposium (IPDPS).*IEEE,2019;314–324. 10.1109/IPDPS.2019.00041

[ref-42] Yang D Zhu Y Wang M : World catalog of Dolichopodidae (Insecta: Diptera). Beijing: China Agricultural University Press,2006. Reference Source

[ref-43] Zhou C McCarthy SA Durbin R : YaHS: yet another Hi-C scaffolding tool. *Bioinformatics.* 2023;39(1): btac808. 10.1093/bioinformatics/btac808 36525368 PMC9848053

