# Supplementary material for: The genome sequence of Dolichopus griseipennis Stannius, 1831
Source: Wellcome Open Res. 2025 Jan 27;10:29. [Version 1] doi: 10.12688/wellcomeopenres.23382.1 (PMC11845904; doi:10.12688/wellcomeopenres.23382.1)
Supplement: Peer review history

# Reviewer report: Reviewer response for version 1

**DOI:** 10.21956/wellcomeopenres.25780.r118366

**Published:** 2025-02-24

**Authors:** Terrence Sylvester

**Affiliations:** 1 University of Memphis, Memphis, TN, USA

The authors have done an excellent work in assembling and annotating the *Dolichopus griseipennis *genome. The authors follow a standard and widely accepted genome sequencing and assembly pipeline. Data are well presented and publicly available. It would be also valuable if the authors assessed the completeness of the annotation using BUSCO or other metric. Other than that, I do not have other significant remarks on the work presented in this manuscript.

Are sufficient details of methods and materials provided to allow replication by others?

Yes

Is the rationale for creating the dataset(s) clearly described?

Yes

Are the datasets clearly presented in a useable and accessible format?

Yes

Are the protocols appropriate and is the work technically sound?

Yes

Reviewer Expertise:

Genomics

I confirm that I have read this submission and believe that I have an appropriate level of expertise to confirm that it is of an acceptable scientific standard.

# Reviewer report: Reviewer response for version 1

**DOI:** 10.21956/wellcomeopenres.25780.r117836

**Published:** 2025-02-21

**Authors:** Carl E Hjelmen

**Affiliations:** 1 Utah Valley University, Orem, Utah, USA

I first want to report that this is my first time reading this manuscript for peer review. In this manuscript the authors are reporting the sequencing and assembling a male of *Dolichopus griseipennis*. This species is beneficial to sequence, given the large number of flies in this genus and family and its rather cosmopolitan distribution. The authors do a great job of highlighting their methods as repeatable and do a nice job of visualizing their data. I believe this work is using the most appropriate methods to result in a quality reference genome. It is clear the work is high quality and is up to standards of data accessibility and quality that we see from the Tree of Life Consortium. I am excited to see this out.

 Below I highlight areas that are of “major” and “minor” concern, with the caveat that all of the comments are minor, but the major ones could improve some aspects of the interpretation of the genome assembly. Many of the concerns on detail I express in the “minor” section below are alleviated with the detail provided in the methods section of this work.

**Major:**

 It is noted that the sequencing methodology used as PacBio SMRT HiFi, but it would be beneficial to report the specific machine used for the sequencing on first report and not just in the methods section.

 The genome was assembled to 897.5 Mbp, but do the authors know how big the genome size of this species is estimated to be or if there is an estimate of the genome size to ensure their assembly is of appropriate size? It is common to have an assembly that is smaller than the size estimated through other methods (such as flow cytometry or Feulgen densitometry)

 The assembly of the genome resulted in 5 autosomal and 1 sex scaffold/pseudochromosome.  Do we know that this species has a karyotype of six chromosomes or is this based on the common haploid numbers of Diptera restricted by Muller elements? I’m not sure how many karyotypes are available for this family of flies (given data available on karyotype.org)

**Minor:**

 The authors do a great job of describing the morphological characteristics of the family, which for a genome paper may not be necessary. That being said, given the nature of the genome report, this seems fine to include to verify the specimen’s taxonomic position and stress the accuracy of its importance and identification.

 The location of the specimen is descriptive but could include a GPS coordinates for more specificity in the first mention of location rather than just in the methods (which is very detailed and appreciated).

Are sufficient details of methods and materials provided to allow replication by others?

Yes

Is the rationale for creating the dataset(s) clearly described?

Yes

Are the datasets clearly presented in a useable and accessible format?

Yes

Are the protocols appropriate and is the work technically sound?

Yes

Reviewer Expertise:

Genome structure, genome size evolution, genome evolution, entomology, phylogenetics

I confirm that I have read this submission and believe that I have an appropriate level of expertise to confirm that it is of an acceptable scientific standard.

# Reviewer report: Reviewer response for version 1

**DOI:** 10.21956/wellcomeopenres.25780.r118364

**Published:** 2025-02-17

**Authors:** Stephen Richards

**Affiliations:** 1 Baylor College of Medicine, Houston, Texas, USA

This is another excellent genome reference sequence by the Darwin tree of life group. The quality is excellent and I can find the data in the databases easily. The data is complete and it's easy to find the transcriptome data too. It's all great news and success for this wonderful project.

Are sufficient details of methods and materials provided to allow replication by others?

Yes

Is the rationale for creating the dataset(s) clearly described?

Yes

Are the datasets clearly presented in a useable and accessible format?

Yes

Are the protocols appropriate and is the work technically sound?

Yes

Reviewer Expertise:

Genomics, Arthropod genomics, dipteran genomics, Drosophila genomics,  human genomics, genome assembly, genome annotation.

I confirm that I have read this submission and believe that I have an appropriate level of expertise to confirm that it is of an acceptable scientific standard.
